# Balloon Retrograde Transvenous Obliteration for Treatment of Persistent Gastric Variceal Bleed in Patient With Hepatocellular Carcinoma

**DOI:** 10.7759/cureus.8796

**Published:** 2020-06-24

**Authors:** Jaimy Villavicencio Kim, Ismail Elkhattib, Daniela Guerrero Vinsard

**Affiliations:** 1 Internal Medicine, University of Connecticut Health Center, Farmington, USA

**Keywords:** brto, gi bleed, gastric varices, hepatocellular carcinoma

## Abstract

Gastric varices are often associated with formation of spontaneous portosystemic shunts that arise to relieve portal hypertension. Balloon retrograde transvenous obliteration (BRTO) is a procedure indicated to prevent recurrent gastric variceal bleeding. Its advantage is diverting blood flow towards the liver, but this can also worsen portal pressure and increase risk for ascites or esophageal variceal bleeding. Patients with gastric varices and concomitant hepatocellular carcinoma (HCC) usually have more advanced cirrhosis and lower possibility of treatment of HCC. BRTO is thought to preserve hepatic function from increased blood flow, possibly allowing better chances of treatment and survival in these patients.

## Introduction

Gastric varices account for 20% of all variceal bleeding, and the one-year risk of bleeding is around 16% [1]. Classification is made according to their location into gastroesophageal varices (GOV1; extending to lesser curvature and GOV2; extending into fundus) and isolated gastric varices (IGV1; in fundus and IGV2; anywhere else in the stomach). They are often associated with formation of spontaneous left-sided portosystemic shunts, which arise to relieve portal hypertension or bypass portal venous obstruction. Gastrorenal shunts are the most common (80%-85%), but direct gastrocaval and gastrocaval via inferior phrenic vein are also commonly seen [2].

Evidence for primary prophylaxis for gastric variceal bleeding is scarce. Only medical management with non-selective beta-blockers is recommended [3]. However, balloon retrograde transvenous obliteration (BRTO) remains one of the first-line therapies to prevent re-bleeding from cardio-fundal varices. During this procedure, a balloon is inserted into the outflow vein of a shunt to allow injection of a sclerosing agent for occlusion. Therefore, careful evaluation of anatomy with CT scan or MRI is required prior to procedure to document presence of a portosystemic shunt. It has the advantage of not diverting blood to bypass the liver, but it can worsen portal pressure and increase risk for ascites or esophageal variceal bleeding [2]. This endovascular technique was perfected and is commonly used in Japan, yet it is not widely practiced in the United States. This is thought to be mainly due to lack of experience with use of sclerosant agents or due to the contradictory method of occluding a spontaneous shunt to relieve portal pressure (which is essentially why transjugular intrahepatic portosystemic shunt [TIPS] is done) [4].

## Case presentation

A 67-year-old male with a past medical history of untreated hepatitis C and hypertension was admitted after experiencing nausea, lightheadedness and a syncopal episode resulting in trauma to his head. En route to the hospital, the patient had one episode of hematemesis, followed by two additional episodes in the emergency department. Vital signs were as follows: heart rate 100 beats per minute, respiratory rate 16 breaths per minute, blood pressure 131/71 mmHg and temperature 94.3°F. The patient was oriented to person, place and time. His abdomen was soft, non-distended and non-tender to palpation. Pertinent laboratory tests included hemoglobin 9.5 g/dl, hematocrit 29.5%, platelets 197 K/µL, blood urea nitrogen (BUN) 22 mg/dl, creatinine 0.8 U/L, aspartate aminotransferase (AST) 112 U/L, alanine aminotransferase (ALT) 76 U/L, total bilirubin 0.5 mg/dl, alkaline phosphatase 18 U/L, albumin 3.2 g/dl, lactic acid 3.1 mmol/L, prothrombin time 11.8 seconds and international normalized ratio (INR) 1.0. Octreotide infusion, ceftriaxone and a proton-pump inhibitor were promptly started, along with transfusion of three units of blood due to persistent bleeding.

**Figure 1:**
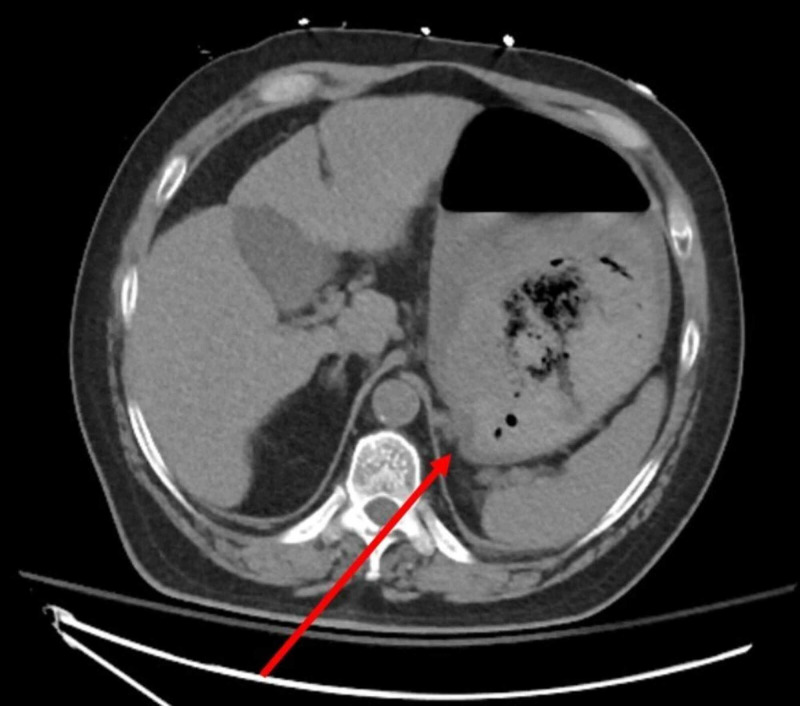
CT scan of the abdomen The red arrow shows gastric varices.

Urgent endoscopy was performed, where a significant amount of red blood was seen in the gastric fundus with an area of active bleeding near the gastroesophageal junction. No esophageal varices were seen. Attempts to control the hemorrhage endoscopically were unsuccessful due to large amount of active bleeding obscuring visibility. CT scan of the abdomen showed a 6-cm mass in the right lobe of a cirrhotic liver (suspected to be hepatocellular carcinoma [HCC]) and one large gastric varix with a gastrorenal shunt (Figure 1). Importantly, there was no evidence of portal vein or splenic vein thrombosis.

**Figure 2:**
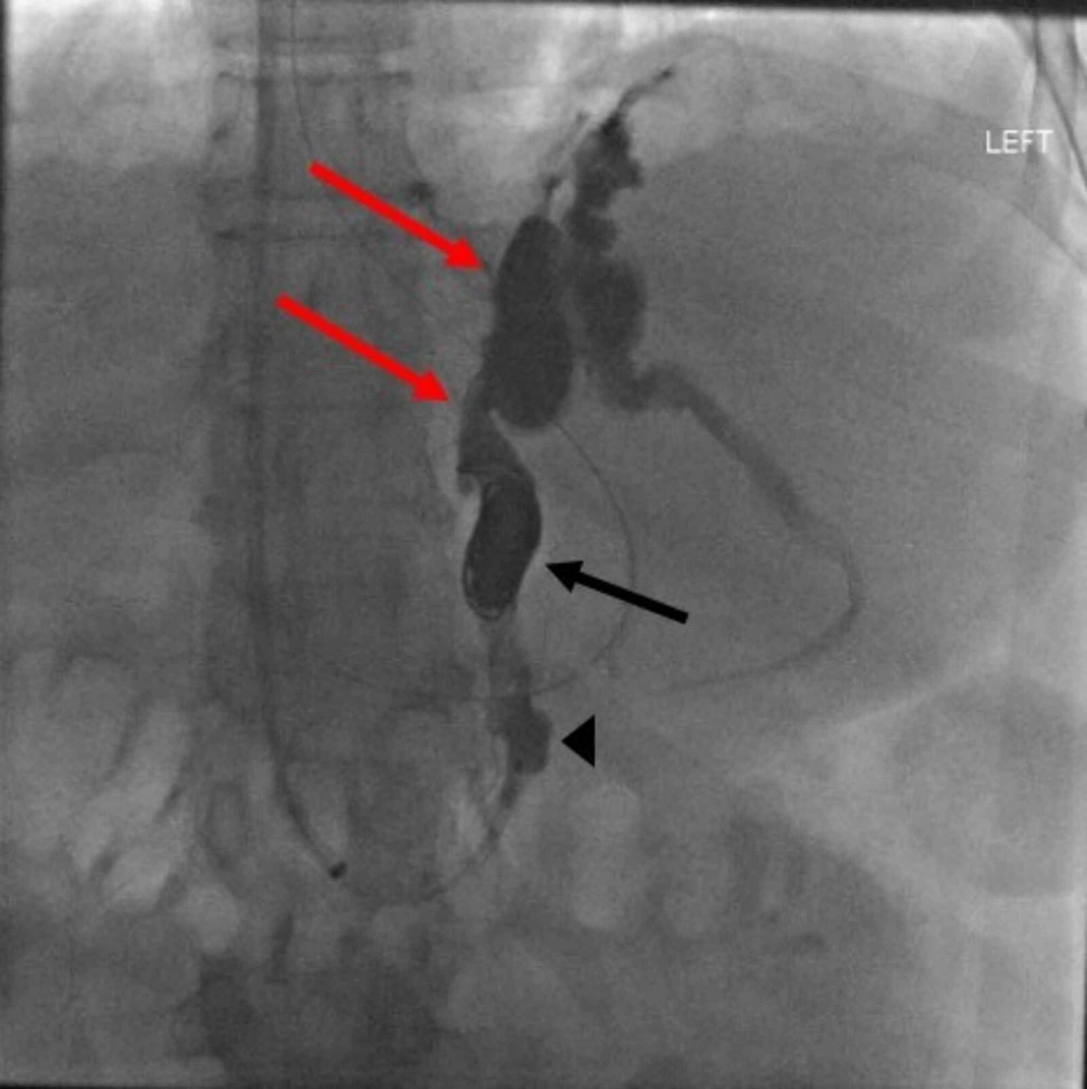
Fluoroscopy image of the BRTO procedure The arrow head points at inflated balloon. The black arrow shows coiling. The red arrow shows gastrorenal shunt. BRTO, balloon retrograde transvenous obliteration.

**Figure 3:**
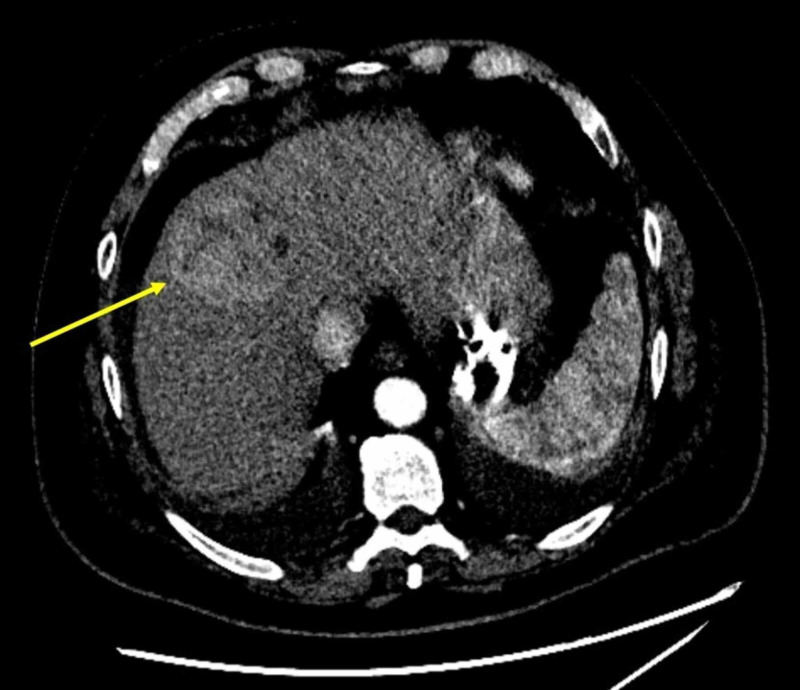
Arterial phase of triple phase CT abdomen The yellow arrow points at hepatocellular carcinoma.

Initially, left-sided TIPS was attempted, but multiple attempts to pass from left portal vein to left hepatic vein were unsuccessful due to challenging anatomy. The patient required multiple blood products. Eventually, the patient underwent BRTO. His left renal vein was accessed via the right internal jugular vein, the balloon was inflated and a sclerosing agent was injected into the bleeding varix. This was followed by coiling of the same varix, resulting in occlusion of the portosystemic shunt (Figure 2). The patient had no further episodes of bleeding and was discharged a few days later. He was ultimately diagnosed with HCC (Figure 3). 

## Discussion

In the American Associated for the Study of Liver Diseases (AASLD) guidelines, BRTO is indicated primarily for prevention of recurrent gastric variceal bleeding [3]. It can be used in patients who are at high risk for TIPS including elevated Model for End-Stage Liver Disease (MELD) score, right-sided heart failure or hepatic encephalopathy. Disadvantages are mainly esophageal variceal bleeding or worsening ascites, as essentially portal pressure worsens with the occlusion of the spontaneous hepatofugal shunt [4]. Contraindications include portal or splenic vein thrombosis without other portosystemic collaterals to provide adequate mesenteric or splenic venous outflow following BRTO [5].

A prospective study showed that patients with gastric variceal bleeding and concomitant HCC usually have more advanced cirrhosis and lower possibility of HCC treatment, due to bleeding-related hepatic decompensation. Another study showed that patients with HCC and esophagogastric varices have overall poorer liver functional reserve [6]. Although BRTO has its disadvantages, its ability to divert blood flow towards the liver is thought to possibly preserve hepatic function. This might make patients with HCC and gastric varices good candidates for BRTO, allowing better possibility for treatment. Further long-term studies are needed to evaluate prognosis and complications of BRTO in these patients.

Patients with variceal bleeding and HCC have worse outcomes compared to those without HCC, and death is most commonly related to hemorrhage. Variceal bleeding is known to be an independent risk factor for overall survival in newly diagnosed HCC and an important component in prognosis [7]. 

## Conclusions

The role of BRTO for primary prevention for gastric variceal bleeding in HCC patients is still obscure. Investigating the benefit of BRTO could be important if there is a possibility of improving survival in these patients. Lack of experience with this procedure could be a limiting factor, but we expect BRTO to be increasingly used in the United States.
